# A funder-imposed data publication requirement seldom inspired data sharing

**DOI:** 10.1371/journal.pone.0199789

**Published:** 2018-07-06

**Authors:** Jessica L. Couture, Rachael E. Blake, Gavin McDonald, Colette L. Ward

**Affiliations:** 1 Bren School of Environmental Science & Management, University of California at Santa Barbara, Santa Barbara, California, United States of America; 2 National Center for Ecological Analysis & Synthesis, University of California at Santa Barbara, Santa Barbara, California, United States of America; 3 School of Marine and Environmental Affairs, University of Washington, Seattle, Washington, United States of America; 4 Marine Science Institute, University of California at Santa Barbara, Santa Barbara, California, United States of America; 5 Department of Evolutionary Biology and Environmental Studies, University of Zürich, Zürich, Switzerland; Tilburg University, NETHERLANDS

## Abstract

Growth of the open science movement has drawn significant attention to data sharing and availability across the scientific community. In this study, we tested the ability to recover data collected under a particular funder-imposed requirement of public availability. We assessed overall data recovery success, tested whether characteristics of the data or data creator were indicators of recovery success, and identified hurdles to data recovery. Overall the majority of data were not recovered (26% recovery of 315 data projects), a similar result to journal-driven efforts to recover data. Field of research was the most important indicator of recovery success, but neither home agency sector nor age of data were determinants of recovery. While we did not find a relationship between recovery of data and age of data, age did predict whether we could find contact information for the grantee. The main hurdles to data recovery included those associated with communication with the researcher; loss of contact with the data creator accounted for half (50%) of unrecoverable datasets, and unavailability of contact information accounted for 35% of unrecoverable datasets. Overall, our results suggest that funding agencies and journals face similar challenges to enforcement of data requirements. We advocate that funding agencies could improve the availability of the data they fund by dedicating more resources to enforcing compliance with data requirements, providing data-sharing tools and technical support to awardees, and administering stricter consequences for those who ignore data sharing preconditions.

## Introduction

In order to enhance discovery, efficiency, and transparency in their work, many scientists have begun to promote the philosophy and practice of open science. Open science advocates a strong focus on data sharing, arguing that publicly available data can have a much greater impact than data that are limited to the creator’s analysis[1–5]. Although this movement has been gaining support and a number of tools have been designed to facilitate openness at each step of the scientific process, those involved in the development of open science acknowledge that the movement is still in its adolescence[4,6–8]. Many researchers are encountering both technical (lack of resources and tools) and cultural (sensitive data, uncomfortable sharing) difficulties in the application of this exciting new approach[4,9–11].

Funding agencies and publishers have long required data sharing or publication of their grantees and accepted submissions. Despite these established policies, weak enforcement has led to consistently low compliance[12–16]. Historically, many technical hurdles to sharing data have existed, and while digitization should help overcome these obstacles, many funders and publishers still fail to provide sufficient infrastructure and technical support for mandated data publication. In addition, an historic culture of perceived ownership of one’s data, combined with an environment of competition for funding and access to publication space, serves as a cultural impediment to the adoption of open data sharing practices[4,17]. In an effort to defend intellectual novelty and guard future publication opportunities, scientists often withhold data from the larger scientific community[18–21].

Although data sharing requirements have been treated with similar leniency by both funders and publishers, the nature of the relationship between a researcher and their funder or publisher may result in different compliance. The funder requirements might be viewed as more urgent, since the funder enabled the data collection and shares some ownership of the data. The publisher merely provides a platform for reporting results, and could therefore be seen to have less authority over the data. Further, publicly funded data is considered a public good and thus should be made available to the entire public[22]. Therefore, researchers might feel more compelled to comply with a funder’s requirements than those of a publisher. Alternatively, since publications are the currency of science, the converse might occur. Several studies have evaluated rates of journal-specific data reporting[12–14,16,23], but none have focused on funder-specific success. There may be differences in the effectiveness of data sharing policies between these funders and publishers. In this study, we sought to determine the recovery rate for a specific funding agency with requirements to make data publicly available.

Data sharing may also be influenced by other characteristics such as the age of data (number of years since data were produced), research field (e.g. oceanographic data, habitat data, social data, etc.), or agency sector of the data creator (e.g. government, private company, native group, etc.). Increasing support for the open science movement would suggest an improved willingness to share data and underscore the hypothesis that more data should be available in recent years than earlier years. Michener et al. (2007) hypothesized a temporal degradation of the knowledge of one’s own data, concluding that as data grow older, less information exists about the data and associated metadata both in the data’s physical form and within the collector’s memory. Recent studies have supported this hypothesis[16,24,25], and as technologies change, these trends are further bolstered by increased availability of data documentation and sharing tools. Larger and faster servers, an increased availability and popularity of collaboration tools such as GitHub, cloud services and free online repositories should lower barriers to data sharing over time [4,7,26]. Similarly, differences in data collection protocols, innovations in instrumentation, or confidentiality of information between research fields may lead to better data preservation in some disciplines but higher hurdles to sharing in others. Furthermore, a scientist’s affiliation may influence willingness or ability to share data. For example, many public government agencies have both external and internal data sharing policies, and are more likely to provide established protocols and systems of data sharing for their employees[3].

We tested the ability to collect ecological and environmental data and evaluate patterns in data recovery for a single funding body. We focused our study on the data-collection of the Exxon Valdez Oil Spill Trustee Council (EVOSTC) funded projects. The EVOSTC was created in 1989 to manage public monetary damages by Exxon Corporation following the Exxon Valdez oil spill in the Gulf of Alaska, and has funded hundreds of research projects since its inception. The EVOSTC requires the public availability of data within one year of data collection for all recipients of their grants, but does not specify archiving methods nor does it provide a specific data publication platform. The EVOSTC did make an effort to collect these data in the mid 1990s, but the success of this effort is unknown as the content of this collection has since been lost. EVOSTC grants have funded work conducted by an array of institutional or agency sectors (government entities, private consulting firms, universities, Alaskan native groups) working in a variety of scientific disciplines. We evaluated 1) How much data could be retroactively recovered and archived? 2) Were there trends in data recovery based on project characteristics? 3) If data were not procured, what were the hurdles to recovery? We also discuss how our results compare to other literature that has examined data sharing and publishing trends, which has focused predominantly on journal publisher requirements.

## Methods

### Data recovery and archiving

From 2012 to 2014, a team of one full-time and three part-time staff was assigned to collect and archive data from projects funded by the EVOSTC, specifically those projects funded between 1989 and 2010. The recovery project was initiated and funded by the EVOSTC for the purpose of recovering and publishing data from EVOSTC funded projects, all of which were conducted under a policy of public ownership of and access to collected data. While the EVOSTC exclusively distributes public funds and has treated its funding as a public good since its inception, there was no formal language requiring public access to collected data until 1994 (S1 Appendix), a factor we account for in our analysis. Project information was obtained from the projects page on the EVOSTC website, which includes varying levels of detail for each project, ranging from only the project title to full bibliographic information and attached reports. Throughout the extensive data recovery effort, careful notes were taken to document and track outreach efforts, communications, and progress in publicly archiving acquired data. Progress was tracked through the data request and acquisition steps for each project based on five stages of increasing progress: “outreach attempted”, “contact established”, “data received”, “published”, “unrecoverable” (Table 1).

**Table 1:** Data recovery status definitions.

Data status	Definition
Outreach attempted	Contact information found and outreach attempted via email and/or phone
Contact established	At least one reply was received by the archiving team from the target researcher. Confirms contact information was correct
Data received	Data was received from the researcher, regardless of data quality or level of documentation
Published	Data were received and documented well enough to be archived by the data team or the researcher cooperated in data clean up and documentation
Unrecoverable	Data were unrecoverable either due to inability to find contact information or for reasons confirmed by the researcher/data owner

Grantee contact information was obtained from agency websites and Google searches based on the information gathered from the EVOSTC website. If contact information was obtained for the listed grantee, an initial outreach email or phone call was made. Attempts were also made to contact additional co-grantees when contact information was available. All contact efforts and communications were tracked on an internal ticketing system. The data support team conducted data outreach and provided data support in the form of data formatting and metadata creation in order to minimize barriers to data sharing. Recovered data were published to the Knowledge Network for Biocomplexity (KNB) data repository. At the close of the data recovery effort (fall 2014), the number of projects was quantified for each of the five status labels (Table 1) to assess the final status of the data recovery effort. Response data were compiled and anonymized for analysis in this project.

### Trends in data recovery

Using the compiled data, each project was characterized based on three project descriptors: *research field of the project* (research field), *agency sector of the home institution of the grantee* (sector), *age of data when recovery efforts were initiated* (age). Within each of these categories a binary response (recovered or not recovered) was used based on the above five status labels, using the following groupings: recovered = “published”, “data received”; not recovered = “unrecoverable”, “outreach attempted”, “contact established”. Since many projects spanned multiple years, age of data was calculated as the number of years since the last year a project received EVOSTC funding. The recovery effort was initiated in 2012 so the most recent data pursued were two years old.

We assessed the impacts of these three project descriptors on the likelihood of providing data using logistic regression analyses[27–32]. We first considered the binary response (data recovered/data not recovered), to test which of our three project descriptors were significant indicators of overall recovery success. We also included a binary factor in each model indicating whether there was a specific requirement of data availability/publication in place to account for inconsistencies in the EVOSTC’s data reporting language. Although the data policy language evolved throughout the funding period, there was no clear way to group the policies beyond presence/absence so a binary variable was used. Since funding for projects was allocated annually (even for multi-year projects), and we consider the procurement of *any* data from a project successful, longer term projects falling during at least one year in which requirements were defined in the funding guidelines were deemed to have had a formal data-sharing policy for the project. A nested logistic regression analysis was then used to assess progress through the recovery process, step-by-step from outreach to publication (Table 1), as in Vines et al. 2014. Each successive step was treated as a binary response conditional on reaching the preceding status level and each binary response variable was used at each step in the recovery process and tested against all three project descriptors.

### Hurdles to recovery

We also examined the reasons data were not recovered. To quantify these reasons we categorized the projects for which we were unable to gather any data based on notes from recovery correspondences (Table 2). The projects included in this part of the analysis were only those labeled “not recovered”. Reasons were only recorded when we had direct confirmation through communications, otherwise projects that were not recovered were characterized based on their status (e.g. the projects from the “outreach attempted” status have been added to the *no contact information* group since we were never able to confirm the outreach efforts actually reached the target recipient). Non-digital data is deemed “unrecoverable” here since this project lacked the resources to convert or store such data during this study.

**Table 2:** Hurdles to recovery category definitions.

Hurdle	Definition
No contact info	No contact information found or contact information was never confirmed because outreach attempts received no response
Communication lost	At least one reply was received by the archiving team from the target researcher but communication was lost before any data were sent
Data lost	PI of other data manager confirmed that the data no longer exist
Non digital data	Data exist in a non-digital format (excludes digital PDFs, includes hand-written or typed data)
Unwilling to share	PI or other data manager refused to share data
Requested funding	PI or other data manager agreed to share data only if additional funding was provided

Data and analyses for this work are archived on the public KNB repository[33]. Tabulated results from the data recovery efforts along with code and results from analyses can be referenced at this URL: https://knb.ecoinformatics.org/#view/doi:10.5063/F1T151VR. No consent was obtained for this work because the data used in this study were anonymized data produced during a previous data recovery effort and therefore could not be linked back to the individual Principal Investigators or data contacts. All data referred to and sought during the initial effort are legally public data; no private data were requested or used for this study (S2 Appendix).

## Results

### Data recovery success

**Fig 1:**
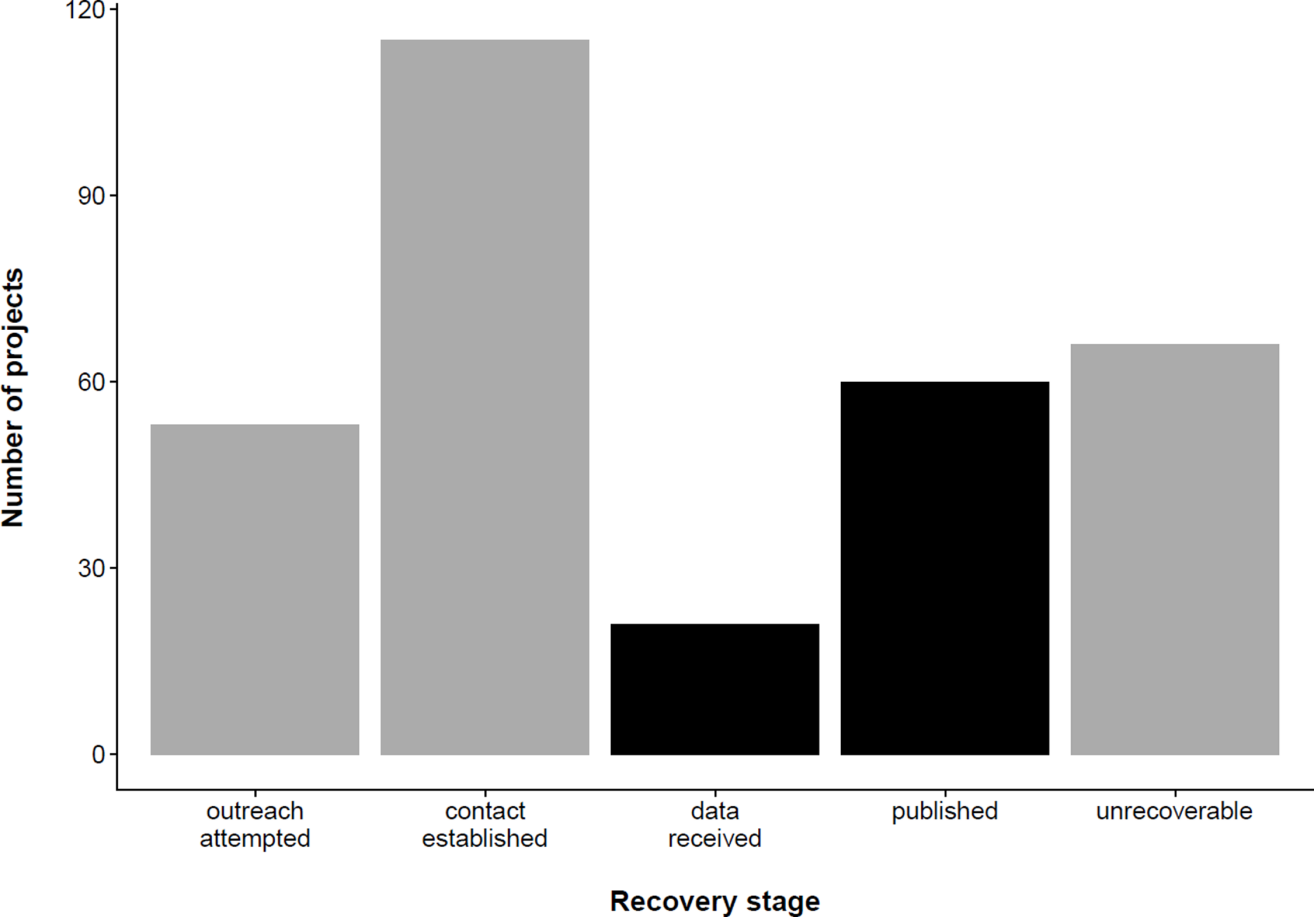
Final status of all data projects. Black bars are projects for which data were successfully acquired, grey bars represent projects for which no data were acquired.

Data were obtained from 81 of the 315 data projects funded from 1989–2010 resulting in recovery of data from 26% of funded projects (Fig 1). Of the 81 projects for which data were recovered, 60 (19% of total, 74% of recovered) provided enough metadata and documentation to create formal archives of the data, while 21 datasets (7% of total) did not include enough information to publish. The team received no data from 234 projects, 23% of which received no reply following outreach and 49% of which lost communication before data were provided (Fig 1). Projects completed after a formal data policy was imposed (data from 1995–2010) had a slightly decreased recovery rate of 24% compared to the overall effort.

### Trends in data recovery

**Fig 2:**
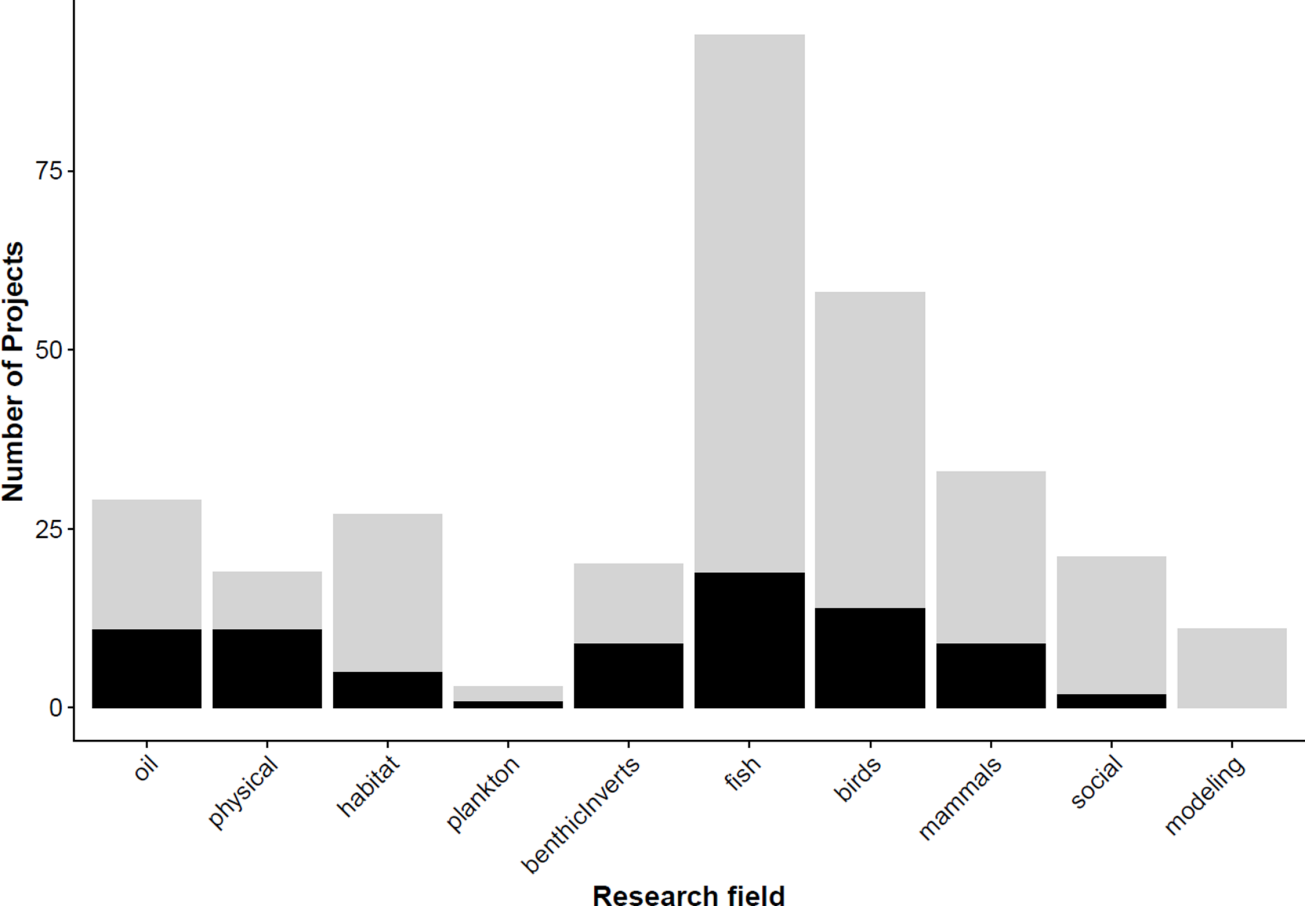
Data recovery grouped by research field. Total successful (black) and unsuccessful (grey) data requests by research field. Asterisks indicate significant research fields.

We examined whether the research field, agency sector, or data age influenced overall recovery of project data, while also accounting for presence of a formal data policy. We saw the highest recovery rates in the fields of physical oceanography (58%) and oil (38%) and the lowest recovery rates in the fields of social science (10%) and modeling (0%), although these were fields with low numbers of projects overall (Fig 2). The greatest number of projects funded was in the biological field, yet we had a low recovery rate for this field (24%). To understand this pattern, we divided the biological field into sub-disciplines and found high variability in recovery rates with the highest recovery rate (45%) for invertebrate-focused projects, and the lowest recovery for habitat-focused (19%), and fisheries-focused (20%) projects, despite fisheries studies being the most numerous (n = 94). Only one field from the research field category, social science, significantly impacted the recovery response (p = 0.0350). No other fields were significant indicators of recovery, although fish and fisheries projects did have low recovery success and is discussed further in the discussion. Sector and age of data each showed high variability (S1 and S2 Figs), but no significant impact on the response. The presence of a formal data policy during the funded project had a significant negative impact on whether data would be recovered (p = 0.0213), indicating that successful recovery decreases when data policies are in place.

The nested analysis assessed the influence of our three project descriptors on progress through the recovery process. Age was found to be a significant indicator of whether contact information was available to initiate initial outreach (first step in data recovery) but did not indicate any further success, or lack thereof, through the recovery process. Neither research field nor agency sector were significant indicators in any of the progress steps. Within the nested analyses, presence of a data policy was a significant indicator of both whether data would be sent once contact was established with a researcher (p = 0.0461) and if data could be published given that data were received (p = 0.0459) but with opposite impacts. Establishment of the data policy had a *negative* impact on whether data would be sent (as with the full, un-nested model) and a *positive* impact on whether data could be published given that they had been received.

### Hurdles to data recovery

**Fig 3:**
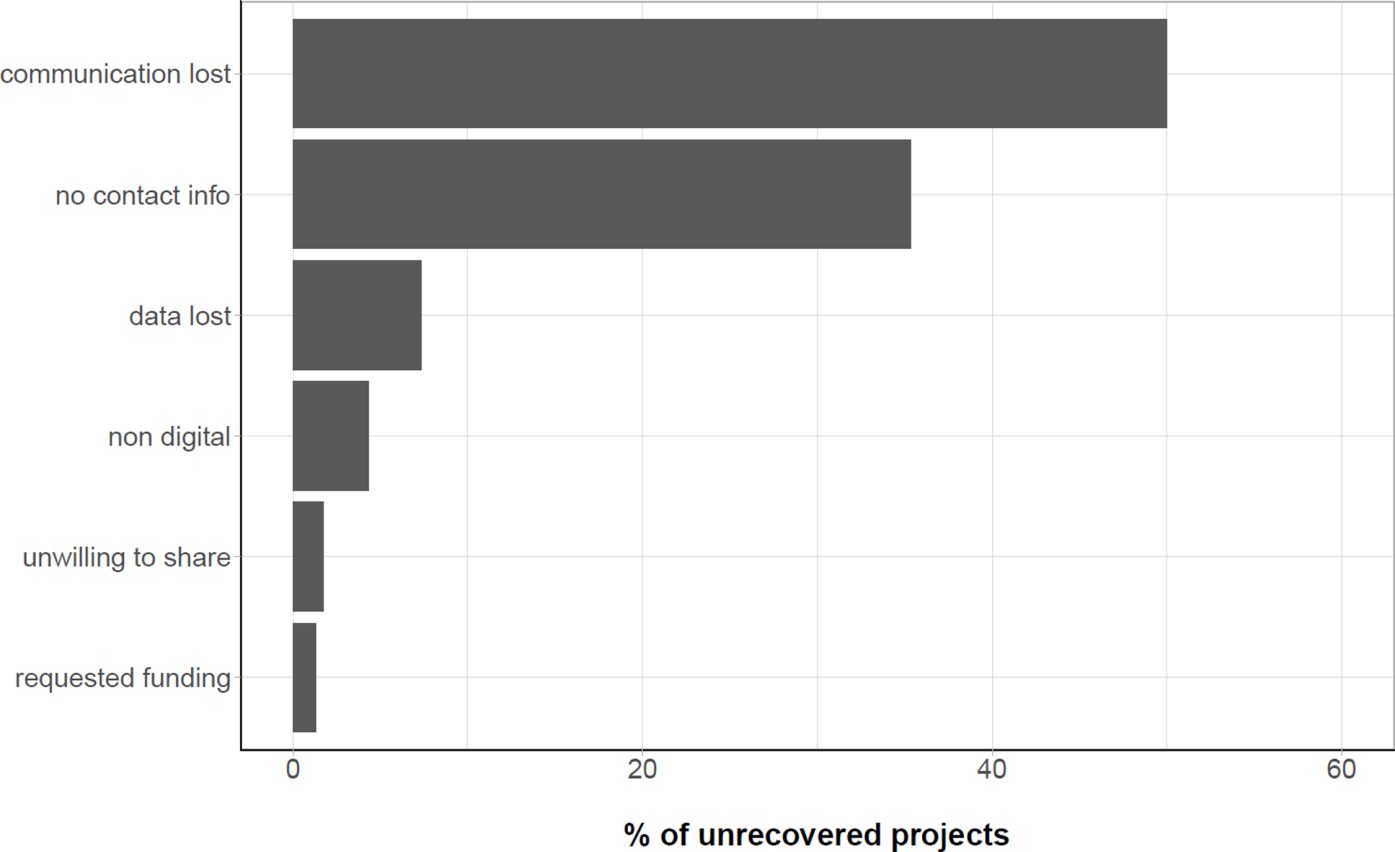
Reasons given for not providing data. Communication loss and lack of contact information were the main reasons data were not obtained.

To examine why our efforts did not recover more data, we examined the frequencies of different types of disruptions in the data recovery process. Loss of communication was the largest hurdle to recovering data. The majority of projects for which data were not recovered became stalled after some initial contact with the grantee had been made, but for which contact was ultimately lost before any data were recovered (50% of non-recovered projects, n = 116) (Fig 3). While loss of communication could be due to reasons that fit under another category, these reasons were not communicated and thus unknown, so these projects were all labeled ‘contact lost’. The second most common hurdle to data recovery was a lack of contact information for grantees or data managers, including projects lacking contact information, as well as those with phone numbers or e-mail addresses from which we did not receive a reply (35%, n = 82) (Fig 3). Data were entirely lost from 7% (n = 17) of the non-recovered projects due to discarded files, obsolete technology, and deceased grantees. We found the frequency of non-digital project data to be quite low (4%, n = 10) and not one of the main factors in our failure to recover data (Fig 3). Grantees specifically requested funding for recovering only 1% (n = 3) of non-recovered projects and flatly refused to share data from only 2% (n = 4) of non-recovered projects. Overall, the biggest hurdle to recovering data remains a loss of communication with the data producers or grantees at all stages of the recovery process.

## Discussion

Overall, we documented a low rate of data recovery, with data successfully recovered from 26% of all funded projects. Of the tested project characteristics, only the research field of the project significantly influenced whether the data would be recovered (Fig 2). In contrast to our expectations, we did not observe a greater recovery rate among more recent projects. However, we did observe a statistically insignificant increase in the fraction of projects for which data recovery was successful in the most recent year studied (S1 Fig), and age of data was an indicator as to whether contact information would be available for the grantee. The presence of formal language requiring data availability had a significant *negative* impact on data recovery success overall. Once data were obtained, the data policy positively impacted whether the data could be published. Recovery rate did not vary with agency sector either, although we similarly had reasons to assume differences would exist (S1 Fig).

The low recovery rate (26%) we obtained is similar to published data recovery rates testing journals with reporting requirements (see Wollins 1962 who reported a 24% recovery rate, Wichert et al. 2006 who reported 26%, Savage & Vickers 2009 who reported 10%, Vines et al. 2014 who reported 19%, Stodden et al. 2018 who reported 44% recovery). In contrast to these studies, we targeted projects funded by a single trustee whose data were *created* under an agreement of public sharing and ownership. While the formal language for this data policy was initially absent (and policy language evolved over the years), all funds were granted under the understanding that data would be made public and the addition of the formal data policy had a negative overall impact on data recovery, likely due to other factors discussed below. We also invested considerably more effort in data recovery in terms of person hours, number of contact attempts, and one-on-one technical support relative to these other studies. Furthermore, although many projects comprised multiple datasets, we considered success to be the receipt of any (but not necessarily all) data associated with a funded project. Therefore, our estimate of 26% recovery is optimistic and likely masks a much lower recovery rate at the dataset level.

This similarity in results suggests that although funders and publishers inherently provide different incentives for data sharing, data-sharing rates are largely unaffected. Funders can be considered to actually share ownership of the data (in this case, the public as well), whereas journals are the gatekeepers of the academic currency of manuscript publication, requiring publication or sharing of data over which they can claim no official ownership. Differentiating between a moral- versus ambition-motivated data sharing impetus appears to be moot as both situations result in similarly low data sharing rates. This result is unsurprising given that both journals and funders have long held these data sharing requirements but rarely enforce compliance and therefore neglect to incorporate adherence into decisions of future benefits (future publications or future funding awards, respectively), an issue we address in depth below. What does differ more than *who* requires data sharing is the field of study in which the data are collected.

The research fields of oil, physical oceanography, plankton, and benthic invertebrates were more likely to share data than other fields. This may be attributable, in part, to the time required to prepare data for sharing. Those research fields that were more likely to share data tend to have more streamlined procedures and data collection processes. Almost all of the oil data sets sought were collected by a single agency, so it was easy to obtain many data sets once communication with that agency was established (of course the opposite would have been true if communication was stalled). Furthermore, these data were likely managed more carefully from the beginning due to the legal relevance of oil records following the oil spill. Meanwhile, both physical oceanography and plankton data sets are often collected using systematic and automated approaches. Once data are collected, they often do not require manual data entry or post-processing and are produced in a final format that is ready to be shared. Previous research indicates data sharing increases with the level of data collection automation[34]. We postulate that researchers may also be likely to share benthic invertebrate data because these are collected using direct observation, using well-established methods. Again, the data are ready to share relatively quickly without post-processing or fear of misinterpretation, thus lowering both operational and personal hurdles to sharing.

The fields that were less likely to share data included fisheries, birds, mammals, habitat, modeling, and social science. These fields tend to collect data that are more sensitive or difficult to interpret, thus leading to concerns about misuse or misinterpretation. Fisheries data are often sensitive because they are frequently used in assessing vulnerable stocks that are fished by diverse groups, while setting management controls such as total allowable catch limits, or in monitoring the catch of sensitive or protected species. This can understandably raise concerns with stakeholders who may worry that sharing their data could reveal prized fishing grounds, result in more restrictive or inequitable management, reveal illegal fishing patterns, or even lead to a fishery or particular areas being closed[35,36]. Meanwhile, the sharing of bird and mammal data faces its own unique challenges due to non-standardization of methods and data complexities. For example, determining abundance in populations with global ranges based on discrete observations takes deep knowledge of the organism and specific population. With no recognized standard for how these data should be structured researchers may be reluctant to share data with scientists unfamiliar with internal protocols[37]. At the same time, researchers studying marine mammals also work with small populations, such as dolphins and sea otters, but with more localized ranges. Researchers may be protective over their research population due to a developed intimacy with their subjects and fear that disclosing their locations could increase their poaching risk[38]. Finally, recovery of social science data shares many of the same obstacles, but many of these datasets also face the additional challenge of involving human subjects. Researchers might fear risking the privacy and confidentiality of their subjects if data are mishandled. Methods do exist to protect against such exposure, but these steps require additional post-processing, inhibiting quick and easy sharing[39–41].

One of our main hypotheses was that data from older projects would be less accessible, but we found limited evidence to support this and we did not observe a gradual decrease in data recovery over time as expected. In addition to expected trends, the EVOSTC documentation did not specifically include any language about data sharing requirements until 1994 (S1 Appendix), despite always acting as a representative of public funds and the data policy language evolved over the 1994–2010 period. Projects ending before 1995 were not more difficult to recover; instead, presence of a data policy negatively impacted data recovery. This result could be an artifact of an early sense of community and collaboration following the EVOS event itself, rather than directly related to the data policy. There were efforts to collect data from projects in the mid-1990s for a 5-year anniversary summary and synthesis of existing results. This collection has since been lost, but the effort may have urged early PIs to organize their data in a shareable format and likely inspired incorporation of formal data policy language in the EVOSTC guidelines. Regardless, it is clear that the presence of the data reporting policy language failed to promote data sharing. We did, however, observe the greatest success rate in the most recent year, in general agreement with Michener's finding that data recovery rates decrease with time since data collection[24]. The expected gradual decline in recovery may have been obscured by two artifacts of our methods: 1) Data recovery rates were based on projects instead of datasets, which may have masked success or failure over time. 2) Since we calculated the “age” of data based on only the last year of the project, few projects happened to end in the most recent years sought, especially since EVOSTC funded projects tended towards longer monitoring and synthesis work in later years (S2 Fig). We did, however, find a significant effect of age on availability of contact information, but once contact information was obtained, there was no relationship between age and researcher response or level of cooperation. Additionally, while there was not a clear impact of age on the ability to publish data given that data were received, there was a significant impact of data policy on data publication in the nested model, suggesting that older projects (pre-data policy) lacked sufficient information to properly document the data and the researcher was unavailable or unable to supply missing material. Therefore, if the Michener temporal trend exists here, the overall trend may have been obscured by methodological details of the data recovery effort.

Inherent in all of the above hurdles to data recovery is the absence of a reward structure for sharing data. There is no reward for the time investment required to learn how to curate data or construct data packages (including well documented and formatted error-free data, with project and file level metadata) in cases where original data curation was insufficient. The focus in science has traditionally been on production and citation of publications, and as such, a process for identification and citation of manuscripts has been well developed. Similar attribution and recognition of *data* would incentivize the archiving and sharing of data in the scientific community. One way publications are tracked and cited is through the use of digital object identifiers (DOIs), a tool that is also increasingly being used to attribute data. DOIs are particularly important for data identification because unlike manuscripts, data can be regularly updated or exist in multiple formats or subsets, so identifying specific versions of the data via a DOI is key to data proper data attribution and use. Although the use of data DOIs is a relatively new practice, they should facilitate more routine data citation as compared to traditional methods. Incorporating data citations into a scientists’ overall research output alongside journal publications should further incentivize data sharing.

Once funding is delivered and papers are published, funding agencies and journals have traditionally neglected to enforce data sharing requirements. These groups could increase compliance by restricting additional funding for violators and providing data tools and assistance to their users [20,42]. For example, the National Science Foundation has created a dedicated data repository, the NSF Arctic Data Center (ADC), where all Arctic data projects funded by NSF must be permanently archived, ensuring proper fulfillment of the data management requirements for their grants. In addition to the infrastructure, the NSF ADC also employs a data support team to assist in data documentation and attribution helping to minimize the work this requirement may add to the grantees’ workloads. NSF enforces these requirements by checking for data archiving in the ADC, or acceptable substitutes, before any additional NSF funding will be granted.

## Conclusion

The open science movement has developed new technologies and worked to move science forward with a culture of data sharing. However, as this study shows, it is still very difficult to obtain data that are required by the funder to be shared. To mitigate data losses, funders and publishers should provide more support and more stringent requirements for data sharing. Incorporating data support and tools into the funding or publication process will help to increase data sharing[4,11]. We found that while the reasons for *not* sharing were uniform across groups, a scientist’s field of research was the strongest indicator of whether they would cooperate to fulfill requests. To address this obstacle, emerging tools should begin to target barriers specific to the fields with lower data-sharing rates. Some of these solutions might include tools that help protect proprietary information, describe methods or facilitate post-processing, and ease the difficulty of preparing data. Finally, personal incentives such as data citations should be more widely used to increase the impact of a particular dataset and provide recognition or credit for data creation. Such improvements are important for increasing the impacts and persistence of data moving forward. Retroactive data archiving projects such as these are essential to capturing and preserving existing data before they are lost. Although the integration of data publication into the scientific process has been slow to be adopted, it is important to increasing efficiency and transparency in science.

## Supporting information

S1 Fig Data recovery by grantee sector. Total successful (black) and unsuccessful (grey) data requests by grantee agency or institution sector.
(EPS)

S2 Fig Data recovery by age of data. Age is calculated based on number of years between the last year of EVOSTC funding and start of the archiving project (2012). Total successful (black) and unsuccessful (grey) data requests by project age.
(EPS)

S1 Appendix History of data requirement documentation from EVOSTC. Chronology of EVOSTC data management policies from 1993–2007.
(PDF)

S2 Appendix Human subjects ethics statement. (PDF)
