# Supplementary material for: A funder-imposed data publication requirement seldom inspired data sharing
Source: PLoS One. 2018 Jul 6;13(7):e0199789. doi: 10.1371/journal.pone.0199789 (PMC6034829; doi:10.1371/journal.pone.0199789)
Supplement: S1 Appendix — Chronology of EVOSTC data management policies from 1993–2007. (PDF) [file pone.0199789.s003.pdf]

Chronology of
EVOS Data Ownership/Management Policies & Projects

March 7, 2007

The following list is the result of a review of annual Invitations for Proposals, Work Plans, and other EVOS TC publications for references to data management policies and projects. Documents are listed by calendar year of publication. Exact wording from the documents appears in quotation marks.

1993

Exxon Valdez Oil Spill Settlement Trustee Council Operating Procedures, January 1993 – no mention of data management.

Draft EVOS Restoration Plan, November 1993, did not contain a reference to data or information, but it did cover public participation. A reference to information and data appears in the final 1994 *EVOS Restoration Plan* [see below under 1994].

Chapter 2 – Polices, page 9:

"8. Meaningful public participation in restoration decisions will be actively solicited."

1994

Invitation to Submit Restoration Projects for Fiscal Year 1995, May 16, 1994.

Chapter 2, page 15: describes the efforts of a working group of agency resource specialists, peer review scientists, and the Public Advisory Group (PAG) to elaborate on the policies in the *Draft Restoration Plan.* The group created Draft Guiding Principles, which were then incorporated into the final *EVOS Restoration Plan.*

Public Participation Principles, page 16:

"11. Restoration must reflect public ownership of the process by timely release and reasonable access to information and data."

EVOS Restoration Plan, November 1994.

Chapter 2 – Mission and Policies

Public Participation, page 17:

"20. Restoration must reflect public ownership of the process by timely release and reasonable access to information and data."

"Information from restoration projects must be available to other scientists and to the general public in a form that can be easily used and understood. An effective restoration program requires the timely release of such information. This policy underscores the fact that since the restoration program is funded with public money, the public owns the results."

Fiscal Year 1995 Work Plan, December 1994:

Administration, Science Management, and Public Information, page 17:

"Project 95089 reflects a major attempt to integrate, synthesize, and make available the information generated by Trustee-sponsored research and restoration activities. It also continues operation of the Oil Spill Public Information Center which has been in existence since 1991. Its FY 95 cost is $522,800."

1995

Invitation to Submit Restoration Projects for Federal Fiscal 1996 and Draft Restoration Program: FY 96 and Beyond, March 24, 1995:

Part II – Public Information/Science Management/Administration:

Information Management System, page 102:

"To improve public access to information generated through the restoration process, in FY 95 the Trustee Council provided funding to develop a plan and the necessary tools to more efficiently synthesize and disseminate information about the oil spill and the Trustee Council in a "user-friendly" manner. The first step in this process is the development of a bibliography of the studies funded by the Trustee Council, which will then be incorporated into an interactive computer program. The information management system will be coordinated with other data management efforts and be made available to the public through OSPIC."

Fiscal Year 1996 Work Plan, December 1995:

Public Information, Science Management, and Administration – funding for OSPIC was separated from funding for the Information Management System.

Information Management System, page 21:

"Beginning in FY 95, the Trustee Council provided funding to develop a comprehensive database of restoration projects and reports for access through the Internet and other necessary tools to more efficiently synthesize and disseminate information generated through the restoration process."

1996

Exxon Valdez Oil Spill Settlement Trustee Council Operating Procedures, adopted August 29, 1996:

Professional Services Contracts

"5. Special Considerations. All notes and other data developed by the contractor shall remain the sole property of the contracting agency."

Invitation to Submit Restoration Proposals for Federal Fiscal Year 1997, February 15, 1996.

Public Information/Science Management/Administration, page 56:

"Project /100 includes funding for:

- Development of a geobibliography of Council-funded databases and an electronic database of all studies funded by the Council."

Procedures for the Preparation & Distribution of Reports, December 1996:

A requirement for a Project Data section on the Study History was added to the procedures with the December 1996 update. The Project Data section was to include the type of data and its location and permanent custodian.

Data Ownership Policy was presented to the TC at their December 6, 1996 meeting. A decision was deferred pending further review. [See the December 6, 1996 EVOS TC meeting transcript, pages 78-86.] A revised policy was approved by the TC at their February 14, 1997 meeting. [See the February 14, 1997 EVOS TC meeting transcript, pages 88-90.] Revisions were based on TC discussion and discussions with attorneys and the EVOS science director and Program Manager. EVOS TC meeting agendas, meeting notes, and a memo from Molly McCammon with the proposed revisions are included at the end of this document.

Fiscal Year 1997 Work Plan, December 1996 – No mention of data management.

1997

A revised *Data Ownership Policy* was approved by the TC at their February 14, 1997 meeting. [See the February 14, 1997 EVOS TC meeting transcript, pages 88-90.] Revisions were based on TC discussion and discussions with attorneys and the EVOS science director and Program Manager. EVOS TC meeting agendas, meeting notes, and a memo from Molly McCammon with the proposed revisions are included at the end of this document.

Invitation to Submit Restoration Proposals for Federal Fiscal Year 1998, February 14, 1997.

If Your Proposal is Funded by the Trustee Council, page 35:

- "Maintain any data recorded during the course of the project and make it available to other researchers and interested parties upon request. The Trust funds are public funds; therefore, all data collected must be accessible to the public."

Fiscal Year 1998 Draft Work Plan, June 1997 – No mention of data management.

1998

Invitation to Submit Restoration Proposals for Federal Fiscal Year 1999, February 15, 1998.

If Your Proposal is Funded by the Trustee Council, page 39:

- "Maintain any data recorded during the course of the project and make it available to other researchers and interested parties upon request. The Trust funds are public funds; therefore, all data collected must be accessible to the public."

Fiscal Year 1999 Draft Work Plan, December 1998 – No mention of data management.

1999

Invitation to Submit Restoration Proposals for Federal Fiscal Year 2000, February 15, 1999.

If Your Proposal is Funded by the Trustee Council, page 43:

- "Maintain any data recorded during the course of the project and make it available to other researchers and interested parties upon request. The Trust funds are public funds; therefore, all data collected must be accessible to the public."

2000

Fiscal Year 2000 Draft Work Plan, January 2000.

Appendix A: Description of Projects and Trustee Council Action

Project 00414-BAA, page A-30

"Ten years after the oil spill there exists a compelling need for translation and communication of scientific results to stakeholders. Interactive web communications can offer a powerful tool for information transfer. This project will develop an architecture and content for interactive, web-based, multimedia delivery of ecosystem research results to the public. The web display will present highlights from the restoration research projects with emphasis on ecosystem synthesis, using a format that is appealing, informative, and understandable. This work will be conducted in

close consultation with Trustee Council staff. Products will reside as a linked modular unit on the Council's website."

Project 00455-BAA, page A-33

"This project will report on the data system issues related to GEM (Gulf Ecosystem Monitoring), the Trustee Council's long-term monitoring and research program. In addition to the data collection effort, data delivery will prove to be a critical component of the success of GEM. Therefore, the data system issues need to be part of the planning process. This project will outline some of the key data and user issues and produce a report analyzing existing systems that deliver similar data. In addition, strawman proposals will be developed for a range of data systems that could meet the needs of the GEM program."

[See the annual report for Project 00455, September 2001, pages 20-29, Background for the GEM Data Policy, and pages 35-54 for a draft of the GEM Program Document Chapter 13: Data Management and Information Transfer.]

Project 00605, page A-44

"Public information is an integral part of Trustee Council activities. This project will increase public awareness and understanding of EVOS restoration activities through improvements to the EVOS web site, improve the ability of researchers to locate and order pertinent publications, and educate managers of fish, wildlife, land, and habitat about new data and new tools available to them through EVOS-funded projects."

Invitation to Submit Restoration Proposals for Federal Fiscal Year 2001, February 2000.

If Your Proposal is Funded by the Trustee Council, page 41:

- "Maintain samples and data taken during the course of the project. By court order, all EVOS-related samples and documents must be retained, with some exceptions. (For more information, see *Procedures for Destroying Documents and Physical Evidence Related to EVOS* available from the Anchorage Restoration Office.) In addition, because Trustee Council funds are public funds, all data collected must be accessible to the public."

[*Procedures for Destroying Documents and Physical Evidence Related to EVOS* can be found at www.evostc.state.ak.us/Proposals/Downloadables/SampleDestruction.pdf]

Gulf Ecosystem Monitoring: A Sentinel Monitoring Program for the Conservation of the Natural Resources of the northern Gulf of Alaska, GEM Science Program NRC Review Draft, April 21, 2000.

Chapter III. Structure and Approach, page 29:

III.G. Data Management, page 36-37:

[See this section for a discussion of data management within the GEM program.]

2001

Fiscal Year 2001 Work Plan, January 2001.

Ecosystem Synthesis/GEM Transition, page 18:

- "Continue data system project (01455-BAA), which in FY 01 will fund a data system manager for GEM."

Appendix A: Description of Projects and Trustee Council Action, page A-26:

01455 – GEM Data System – Abstract:

"This project will initiate an ongoing data system for GEM (Gulf Ecosystem Monitoring, the Trustee Council's long-term monitoring and research program currently under development). GEM is being designed to monitor the ecosystems of the northern Gulf of Alaska and the adjacent coastal regions for a very long time period. Data collection, archiving, transfer, delivery, and presentation are critical components of GEM. FY 01 funding will be used to hire a data system manager to provide the leadership necessary for developing this essential part of the GEM program."

Project 00455 Annual Report, September 2001:

- pages 20-29 for Background for the GEM Data Policy and
- pages 35-54 for a draft of the GEM Program Document Chapter 13: Data Management and Information Transfer.

Invitation to Submit Restoration Proposals for Federal Fiscal Year 2002, February 2001.

Ecosystem Synthesis/GEM Transition, page 30:

Potential Continuing Projects:

"Data System for Gem (/455) In FY01 this project provided funding for a data systems manager for GEM. Efforts in FY 01 (hiring is expected in June 2001) will focus on system design. Efforts in FY 02 and beyond will include collaboration with Trustee agencies and other data systems as well as data input, linking and management."

If Your Proposal is Funded by the Trustee Council, page 39:

- "Maintain samples and data taken during the course of the project. By court order, all EVOS-related samples and documents must be retained, with some exceptions. (For more information, see *Procedures for Destroying Documents and Physical Evidence Related to EVOS* available from the Anchorage Restoration Office.) In

addition, because Trustee Council funds are public funds, all data collected must be accessible to the public."

[*Procedures for Destroying Documents and Physical Evidence Related to EVOS* can be found at www.evostc.state.ak.us/Proposals/Downloadables/SampleDestruction.pdf]

Fiscal Year 2002 Work Plan, December 2001.

Data Management & Information Transfer, page 15:

- "Continue GEM data system project (02455), which in FY02 will fund a data manager for GEM."

Appendix A: Description of Projects and Trustee Council Action, page A-22:

02455 – GEM Data System – Abstract:

"This project will continue work on the data system for GEM by hiring a data system manager to provide the leadership necessary for developing this essential part of the GEM system."

2002

Invitation to Submit Restoration Proposals for Federal Fiscal Year 2003, Phase I, February 2002.

Data Management & Information Transfer, page 15:

"Data System for GEM (/455). In FY 02, this project provided funding for a data systems manager for GEM. Efforts in FY 02 (hiring is expected in February 2002) will focus on development of a data policy for GEM and on system development and design. Efforts in FY 03 and beyond will include collaboration with Trustee agencies and other data systems as well as data input, linking, and management. This project is in the "potential continuing projects" category because its actual scope and cost for FY 03 is not yet known."

If Your Proposal is Funded by the Trustee Council, page 27:

- "Maintain samples and data taken during the course of the project. By court order, all samples and documents must be retained, with some exceptions. Because EVOS funds are public funds, all data collected must be available to the public. (Contact the Anchorage Restoration Office for a copy of the Trustee Council's sample destruction and data policies.) Anew data policy, under development for GEM, will likely apply to all FY 03 projects. All prospective EVOS investigators will be advised of the new data policy prior to Phase I funding awards."

Gulf of Alaska Ecosystem Monitoring and Research Program (GEM): The GEM Program Document, July 9, 2002.

Chapter 9. Data Management and Information Transfer, page 341-356.

[See this section for a detailed discussion of data management within the GEM program.]

Invitation to Submit Restoration Proposals for Federal Fiscal Year 2003, Phase II, July 2002.

Attention All Proposers: New Data Policy & Procedures, page 3:

"For those of you who have participated in the Trustee Council's restoration program in the past, please note that the Council has adopted a new data policy and revised its project report requirements. The data policy and the report procedures are available from the Trustee Council Office or on the web at:
http://www.oilspill.state.ak.us/pdf/admin/datapolicy.pdf (data policy)
http://www.oilspill.state.ak.us/pdf/admin/reportguidelines.pdf (report procedures)"

If Your Proposal is Funded by the Trustee Council, page 16:

"During project implementation, principal investigators (PIs) must do the following:

- "Develop a data management plan. In collaboration with the Trustee Council's Data Systems Manager, develop a data management plan. This plan will include procedures to process, document and migrate all data to be collected to archives identified by the Data Systems Manager. In addition, the Data Systems Manager will collaborate with PIs on data formats. (For more information, see *Data Policy* available from the Trustee Council Office or on the web at http://www.oilspill.state.ak.us/pdf/admin/datapolicy.pdf."

- "Maintain samples and data taken during the course of the project. Because the Trustee Council's program is still subject to potential litigation, certain requirements have been imposed by state and federal courts regarding destruction of samples and documents related to EVOS. There are significant legal consequences if items are destroyed other than a prescribed by the courts. (For more information, see Procedures for Destroying Documents or Physical Evidence Related to EVOS available from the Trustee Council Office or on the web at http://www.oilspill.state.ak.us/admin/prosample.pdf."

Exxon Valdez Oil Spill Trustee Council General Operating Procedures, adopted July 9, 2002, revised October 4, 2006. page II-4

Project Data

"1. *Metadata and Data.* During the course of the project and at its completion, the investigator shall submit metadata ("data about data") and project data according to Trustee Council approved data policies. The metadata and project data contents, format, and review procedures shall be determined by the Executive Director."

A Century of Ecosystem Science: Planning Long-Term Research in the Gulf of Alaska, National Research Council, National Academy Press, Washington, D.C. 2002.

Data management is discussed on page 8, page 83, and in Chapter 6 – Data and Information Management., page 66-68. Page 68, last paragraph discusses the importance of having a "clear, established data policy and a willingness to enforce it."

Fiscal Year 2003 Work Plan, November 2002.

Data Management & Information Transfer, page 20:

- "Continue GEM data system project (G-030455), which funds the GEM Data Systems Manager and related data system costs such as quality control and documentation, archiving, transfer, delivery and presentation."

Appendix A: Description of Projects and Trustee Council Action, page A-8:

G-030455 – GEM Data System – Abstract:

"This project supports the data management and information transfer system for GEM. Data collection, quality control and documentation, archiving, transfer, delivery, and presentation are critical components of GEM. Project funding will allow the GEM Data Systems Manager to provide the leadership necessary for this essential part of the GEM program, and hire support staff to make initial aspects of the program operational."

2003

Gulf of Alaska Ecosystem Monitoring and Research Program, Draft FY 2004 Work Plan, August 22, 2004. [Note: no project numbers are used in this work plan.]

Data Management, page 17-18. See discussion under Introduction.

[Did not review FY 04 Invitation – no copy available at ARLIS or on the EVOS TC website.]

2004

Gulf of Alaska Ecosystem Monitoring and Research Program, Work Plan FY 2004 – FY 2006, January 9, 2004. [Note: no project numbers are used in this work plan.]

Data Management, page 24. [See discussion under Introduction.]

Invitation to Submit Proposals: Federal Fiscal Year 2005, March 2, 2004.

Summary of Invitation for All Program Areas:

Data Management and Information Transfer, page 3:

"Proposals are invited to construct a database of metadata describing real time sensors from the northern Gulf of Alaska relevant to GEM."

Data Management and Information Transfer, page 5-6.

[See this section for a description of the definition and uses of Data Management within GEM.]

General Condition, page 18:

- "Develop a data management plan. In collaboration with the Trustee Council's Data Systems Manager, develop a data management plan that includes procedures to process, document and migrate all data to be collected to archives identified by the Data Systems Manager. In addition, the Data Systems Manager will collaborate with PIs on data formats. For more information, see *Data Policy* at http://www.oilspill.state.ak.us/pdf/admin/datapolicy.pdf."

- "Maintain samples and data taken during the course of the project. Because the Trustee Council's program is funded by a court-approved settlement with Exxon Corp., it is still subject to potential litigation. Certain requirements have been imposed by state and federal courts regarding destruction of samples and documents related to EVOS. There are significant legal consequences if items are destroyed other than a prescribed by the courts. (For more information, see Procedures for Destroying Documents or Physical Evidence Related to EVOS available from the Trustee Council Office or on the web at http://www.oilspill.state.ak.us/admin/prosample.pdf."

How to Prepare a Proposal, page 22-25:

[This section discussed data management requirements. The starting page may be 22 or 23, and cannot be verified because those pages are missing from the print document.]

Exxon Valdez Oil Spill Trustee Council Work Plan FY 2005 – FY 2007, October 8, 2004.

Data Management, page 53. See discussion under Introduction.

Table of Data Management Projects:

"There are no external Data Management projects funded in FY 2005."

2005

Invitation for Proposals: Federal Fiscal Year 2006, February 11, 2005.

General Conditions, page 19:

"During project implementation, principal investigators (PIs) must do the following:

- "Develop a data management plan. In collaboration with the Trustee Council's Data Systems Manager, develop a data management plan

that includes procedures to process, document and migrate all data to be collected to archives identified by the Data Systems Manager. In addition, the Data Systems Manager will collaborate with PIs on data formats. For more information, see *Data Policy* at http://www.oilspill.state.ak.us/pdf/admin/datapolicy.pdf."

How to Prepare a Proposal, page 23-24:

Data Management and Quality Assurance/Quality Control (QA/QC) Statement:

[See this section for detailed instructions and requirements.]

2006

Invitation for Proposals: Federal Fiscal Year 2007, June 1, 2006.

Data Management and Synthesis, page 19:

"The Council will consider proposals that facilitate recovery, utilization and/or enhancement of long-term data series within the oil spill affected areas. Information should assist the Council in identifying appropriate restoration projects and determining recovery of injured resources or service."

Data Management and Quality Assurance/Quality Control (QA/QC) Statement, page 23-25:

[See this section for detailed data management requirements.]

11.8.2 K

Exxon Valdez Oil Spill Trustee Council

Restoration Office

645 G Street, Suite 401, Anchorage, Alaska 99501-3451

Phone: (907) 278-8012 Fax: (907) 276-7178

TRUSTEE COUNCIL MEETING ACTIONS

RECEIVED
FEB [illegible] 1997
EXXON VALDEZ OIL SPILL
TRUSTEE COUNCIL
ADMINISTRATIVE RECORD

DRAFT

December 6, 1996 @ 10 a.m.

By Molly McCammon
Executive Director

Trustee Council Members Present:

- •Jim Wolfe, USFS
- •Deborah Williams, USDOI
- •Bill Hines, NMFS
- •Janet Kowalski, ADF&G
- •Michele Brown, ADEC
- *•Craig Tillery, ADOL

* Chair

- Alternates:

  Janet Kowalski served as an alternate for Frank Rue for the entire meeting.
  Al Ewing served as an alternate for Michele Brown for a portion of the meeting.
  Bill Hines served as an alternate for Steve Pennoyer for the entire meeting. Steve Pennoyer was present during the Executive Session.
  Jim Wolfe served as an alternate for Phil Janik for the entire meeting. Phil Janik was present during the Executive Session.
  Deborah Williams served as an alternate for George T. Frampton, Jr. for the entire meeting.
  Craig Tillery served as an alternate for Bruce Botelho for the entire meeting.

1. Approval of the Agenda

APPROVED MOTION: Approved the Agenda. Motion by Williams, second by Brown.

2. Approval of the Meeting Minutes

APPROVED MOTION: Approved November 8, 1996 Trustee Council meeting notes. Motion by Williams, second by Hines.

3. Natural Resources Damage Assessment Reports

APPROVED MOTION: Adopted option number 4 - to address what to do with NRDA projects without a final report on a case by case basis and report back to the Trustee Council within six months on their status. Motion by Williams, second by Brown.

4. Archaeology Planning Project

APPROVED MOTION: Approved $12,100 to print additional Archaeology Planning Reports, postage for the distribution of the reports and for three staff members to travel to the communities to hold public meetings and develop further options for Council consideration. Motion by Williams, second by Brown.

5. Restoration Reserve Plan

APPROVED MOTION: Approved Executive Director's recommendation to begin public planning process on Restoration Reserve.

6. Public Comment Period

Eight members of the public testified from Anchorage and one individual testified from Homer.

7. Executive Session

APPROVED MOTION: Adjourn into Executive Session to discuss Habitat Protection, Public Advisory Group nominations, the Executive Director's evaluation, and the Exxon settlement re-opener clause. Motion by Wolfe, second by Brown.

(Off Record at 12:04 p.m.)
(On Record at 1:35 p.m.)

8. Public Advisory Group Nominations

APPROVED MOTION: Nominated the following individuals to sit on the Public Advisory Group for the 1997 - 1998 term:

Mary McBurney - Aquaculture
Torie Baker - Commercial Fishing
Eleanore Huffines - Commercial Tourism
Chip Dennerlein - Conservation
Pam Brodie - Environmental
Howard Valley - Forest Products
Dave Cobb - Local Government
Chuck Totemoff - Native Landowner
Stacey Studebaker - Recreation Users
Rupert Andrews - Sport Hunting and Fishing
Nancy Yeaton - Subsistence
Chuck Meacham - Science/Academic
Chris Beck - Public-at-Large
Vern McCorkle - Public-at-Large

Sheri Buretta - Public-at-Large
Jim King - Public-at-Large
Brenda Schwantes - Public-at-Large
Motion by Hines, second by Ewing.

DRAFT

9. Traditional Ecological Knowledge Protocols

APPROVED MOTION: Adopt the Executive Director's recommended guidelines for including indigenous knowledge in the restoration process. Motion by Williams, second by Hines.

10. Data Ownership and Archiving Policy

APPROVED MOTION: Deferred action on policy pending further review.

11. 1997 Deferred Project Proposals

APPROVED MOTION: Adopt the Executive Director's recommendations on the deferred project proposals for 1997 totaling $609,200. Request additional information on Project 97254, Delight and Desire Lakes Fertilization. Motion by Williams, second by Ewing.

12. Tatitlek

APPROVED MOTION: Adopt the revised Tatitlek resolution accepting Tatitlek Corporation's counteroffer of $33,800,000 (from $33,000,000) for various interests in 66,000 acres plus a timber only conservation easement on the Sunny Bay parcel (approximately 2,445 additional acres). Motion by Wolfe, second by Williams.

13. Horseshoe Bay/PWS 11

APPROVED MOTION: Authorize the Alaska Department of Natural Resources to acquire the Horseshoe Bay parcel (PWS 11), consisting of 1,600 feet of Horseshoe Bay frontage and including the mouth of an anadromous stream, for the approved appraisal price of $475,000. Motion by Wolfe, second by Kowalski.

14. Johnson Parcel KAP 114

APPROVED MOTION: Authorize the U.S. Fish and Wildlife Service to acquire the Johnson Parcel (KAP 114), consisting of 55 acres located within Uyak Bay for the approved appraisal price of $154,000. Motion by Williams, second by Hines.

15. Kenai Natives Association Small Parcels

APPROVED MOTION: Adopt a motion clarifying that with the KNA acquisition, the state is to receive conservation easements only with respect to the Stephanka and Moose River patented tracts. Motion by Williams, second by Ewing.

16. Coordination with Native Groups at the Alaska SeaLife Center

APPROVED MOTION: Encourage the Executive Director of the Alaska SeaLife Center "to work closely with the local Native community on the issues including, consideration of the request for formation of a committee, or other working group, for the purpose of assisting the SeaLife Center in producing a respectful and accurate representation of the traditional heritage of Alaskan Native Culture." Motion by Williams, second by Ewing.

Meeting adjourned at 3:17 p.m.

DRAFT

raw

11.8.2G

Exxon Valdez Oil Spill Trustee Council

645 G Street, Suite 401, Anchorage, AK 99501-3451 907/278-8012 fax: 907/276-7178

RECEIVED
FEB 1[illegible] 1997
EXXON VALDEZ OIL SPILL
TRUSTEE COUNCIL
ADMINISTRATIVE RECORD

MEMORANDUM

To: Gina Belt, Maria Lisowski, Barry Roth, and Alex Swiderski

From: Molly McCammon, Executive Director

Date: February 6, 1997

Subject: Data Ownership Policy

Please find enclosed a revised version of the proposed data ownership policy for the Trustee Council. This reflects the discussion at the December 6, 1996 Trustee Council meeting as well as more recent discussions among Barry Roth, Alex Swiderski, Eric Myers, and Stan Senner. I intend to bring this up for action at the Trustee Council meeting on February 14, the packets for which will be distributed tomorrow, February 7. If there are any additional concerns that need immediate attention, please let Eric or Stan know today.

encl: (1)

Federal Trustees
U.S. Department of Interior
U.S. Department of Agriculture
National Oceanic and Atmospheric Administration

State Trustees
Alaska Department of Fish and Game
Alaska Department of Environmental Conservation
Alaska Department of Law

For Consideration at the Trustee Council Meeting February 14, 1997

EXISTING POLICY

According to policy number 20 in the Exxon Valdez *Oil Spill Restoration Plan* (November 1994):

> **Restoration must reflect public ownership of the process by timely release and reasonable access to information and data.**
>
> Information from restoration projects must be available to other scientists and to the general public in a form that can be easily used and understood. An effective restoration program requires the timely release of such information. This policy underscores the fact that since the restoration program is funded by public money, the public owns the results.

In addition, item number 5 under Professional Services Contracts in the Trustee Council *Procedures* adopted August 29, 1996 states:

> *Special Considerations.* All notes and other data developed by the contractor shall remain the sole property of the contracting agency.

PROPOSED CLARIFICATION

We now propose to clarify this statement of Trustee Council policy by adoption of the following:

> Therefore, consistent with state and federal laws, any data ~~or other products~~ resulting from any project to which the Trustee Council has contributed financially are in the public domain and as such must be available to the public. Fees will only be charged for copies of data in accordance with the Federal Freedom of Information Act, the State Public Records Act, or other applicable law. Data means recorded information, regardless of form or the media on which it is recorded, including computer programs, data bases, and software. Each final report on a restoration project shall include a brief description of data gathered in the project, including definition of the types of data gathered, the form or forms in which the data are recorded, the location of the data, and a permanent contact at ~~a public institution~~ the appropriate federal or state agency such that the data are accessible to the public, including scientific users, after completion of the project.

11.8.2 L

Exxon Valdez Oil Spill Trustee Council

Restoration Office

645 G Street, Suite 401, Anchorage, Alaska 99501-3451

Phone: (907) 278-8012 Fax: (907) 276-7178

RECEIVED
FEB 10 1997
[illegible] Information Center
645 G Street
Anchorage, AK 99501

RECEIVED
FEB [illegible] 1997
EXXON VALDEZ [illegible]
TRUSTEE COUNCIL
ADMINISTRATIVE RECORD

2/7/97
1:18 pm
DRAFT

AGENDA
EXXON VALDEZ OIL SPILL SETTLEMENT
TRUSTEE COUNCIL CONTINUATION MEETING

FEBRUARY 14, 1997 @ 8:30 A.M.

645 G STREET, ANCHORAGE

Trustee Council Members:

BRUCE BOTELHO/CRAIG TILLERY
Attorney General/Trustee
State of Alaska/Representative

MICHELE BROWN
Commissioner
Alaska Department of Environmental Conservation

GEORGE T. FRAMPTON, JR./DEBORAH WILLIAMS
Assistant Secretary/Trustee Representative for Fish & Wildlife & Parks
U.S. Department of the Interior

PHIL JANIK
Regional Forester - Alaska Region
U.S. Department of Agriculture
Forest Service

STEVE PENNOYER
Director, Alaska Region
National Marine Fisheries Service

FRANK RUE
Commissioner
Alaska Department of Fish & Game

Teleconferenced in Juneau, LIO, Goldstein Building, Room 319
Craig Tillery, Chair

1. Call to Order 8:30 a.m.
   - Approval of Agenda
   - Approval of December 6, 1996 meeting notes

2. Executive Director's Report - Molly McCammon
   - Administrative Issues
     - Office Space
   - Quarterly Project Status Report
   - Quarterly Financial Status Report
   - Status of Audit
   - FY98 Invitation and Work Plan
   - Other

3. Public Comment - 9 a.m.

4. Status Report on the Archaeological Planning Effort

Trustee Agencies
State of Alaska: Departments of Fish & Game, Law, and Environmental Conservation
United States: National Oceanic and Atmospheric Administration, Departments of Agriculture and Interior

5. Data Policy*

6. Deferred Work Plan Projects
   - Amendment to 97100 for Video Production*
   - Project 97162 - Herring Disease*
   - Project 97248 - Historical Data and Local TEK - Herring*
   - Project 97254 - Delight/Desire Lake*

7. Recognition of George T. Frampton, Jr. and Doug Hall

8. Executive Session on Habitat and Executive Director's Evaluation

9. English Bay Acquisition*

10. Small Parcel Program*
    - Status Report
    - KEN 1038 - Roberts (Schilling) Parcel

* indicates tentative action items

Adjourn - 1:30 p.m.

raw

For Consideration at the Trustee Council Meeting February 14, 1997

EXISTING POLICY

According to policy number 20 in the Exxon Valdez *Oil Spill Restoration Plan* (November 1994):

> **Restoration must reflect public ownership of the process by timely release and reasonable access to information and data.**
>
> > Information from restoration projects must be available to other scientists and to the general public in a form that can be easily used and understood. An effective restoration program requires the timely release of such information. This policy underscores the fact that since the restoration program is funded by public money, the public owns the results.

In addition, item number 5 under Professional Services Contracts in the Trustee Council *Procedures* adopted August 29, 1996 states:

> *Special Considerations.* All notes and other data developed by the contractor shall remain the sole property of the contracting agency.

PROPOSED CLARIFICATION

We now propose to clarify this statement of Trustee Council policy by adoption of the following:

> Therefore, consistent with state and federal laws, any data ~~or other products~~ resulting from any project to which the Trustee Council has contributed financially are in the public domain and as such must be available to the public. Fees will only be charged for copies of data in accordance with the Federal Freedom of Information Act, the State Public Records Act, or other applicable law. Data means recorded information, regardless of form or the media on which it is recorded, including computer programs, data bases, and software. Each final report on a restoration project shall include a brief description of data gathered in the project, including definition of the types of data gathered, the form or forms in which the data are recorded, the location of the data, and a permanent contact at ~~a public institution~~ the appropriate federal or state agency such that the data are accessible to the public, including scientific users, after completion of the project.

11.8.3 t

Exxon Valdez Oil Spill Trustee Council

Restoration Office
645 G Street, Suite 401, Anchorage, Alaska 99501-3451
Phone: (907) 278-8012 Fax: (907) 276-7178

TRUSTEE COUNCIL MEETING ACTIONS

February 14, 1997 @ 8:30 a.m.

DRAFT

By Molly McCammon
Executive Director

Trustee Council Members Present:

•Jim Wolfe, USFS	Frank Rue, ADF&G
•Deborah Williams, USDOI	Michele Brown, ADEC
Steve Pennoyer, NMFS	*•Craig Tillery, ADOL

* Chair
• Alternates:
Deborah Williams served as an alternate for George T. Frampton, Jr. for the entire meeting.
Jim Wolfe served as an alternate for Phil Janik for the entire meeting.
Craig Tillery served as an alternate for Bruce Botelho for the entire meeting.

1. Approval of the Agenda

APPROVED MOTION: Approved the Agenda. Motion by Williams, second by Wolfe.

2. Approval of the Meeting Minutes

APPROVED MOTION: Approved December 6, 1996 Trustee Council meeting notes. Motion by Pennoyer, second by Rue.

3. Data Policy

APPROVED MOTION: Adopted the Data Policy detailed as an attachment to the February 6, 1997 memo from the Executive Director to state and federal attorneys. Motion by Williams, second by Rue. (Attachment A)

4. Deferred Work Plan Projects (Attachment B)

APPROVED MOTION: Adopted the Executive Director's recommended amendment to Project 97100, that adds $71,400 in FY97 funds and $29,300 in FY98 funds to obtain documentary-style film and still photos. Motion by Williams, second by Rue.

Trustee Agencies
State of Alaska: Departments of Fish & Game, Law, and Environmental Conservation
United States: National Oceanic and Atmospheric Administration, Departments of Agriculture and Interior

APPROVED MOTION: Adopted the Executive Director's recommendation on Project 97162 for an additional $34,300 to study the disease levels associated with the herring pound fishery in Prince William Sound. Motion by Rue, second by Brown.

APPROVED MOTION: Adopted the Executive Director's recommendation on Project 97248 to provide $46,900 in funds and to integrate it into SEA Project 97320T, contingent upon an approved detailed project description and budget. Motion by Williams, second by Rue.

APPROVED MOTION: Adopted the Executive Director's recommendation on Project 97254 to approve the project for $123,100 for limnological work only and that the Alaska Department of Fish and Game work cooperatively with the Department of the Interior to carry out this project. Motion by Rue, second by Wolfe.

5. Executive Session

APPROVED MOTION: Adjourn into executive Session for the purpose of discussing habitat acquisition and the Executive Director's evaluation. Motion by Williams, second by Rue.

Off Record at 11:24 a.m.
On Record at 1:05 p.m.

6. English Bay

APPROVED MOTION: Approved resolution to offer English Bay Corporation $14.1 million for approximately 32,400 acres. Motion by Williams, second by Rue. (Attachment C)

7. Roberts Parcel (KEN - 1038)

APPROVED MOTION: Approved resolution to offer $698,000 for 3.34 acres on the Kenai River, in Soldotna, for the Roberts/Schilling parcel. Motion by Rue, second by Brown. (Attachment D)

8. Chenega Amendment

APPROVED MOTION: Approved the amended Chenega resolution clarifying that of the $34,000,000 purchase price, $24,000,000 comes from the Trustee Council and $10,000,000 from the federal restitution funds. Second, it allows the Trustee Council to go forward with the court

request for the required funds and deposits the money into the federal NRDA R account to be immediately available for closing. Motion by Williams, second by Wolfe. (Attachment E)

Meeting recessed at 1:37 p.m.

raw
