# Supplementary material for: A funder-imposed data publication requirement seldom inspired data sharing
Source: PLoS One. 2018 Jul 6;13(7):e0199789. doi: 10.1371/journal.pone.0199789 (PMC6034829; doi:10.1371/journal.pone.0199789)
Supplement: S2 Appendix — (PDF) [file pone.0199789.s004.pdf]

S2 Appendix. Human subjects ethics statement.

No consent was obtained for this work because human subjects were not used in this work. The data used in this project focused solely on the projects and datasets themselves, and specifically the results of the previous data recovery effort. No direct or indirect contact or communication occurred between the authors and dataset Principal Investigators during this project. There was no solicitation or use of Principal Investigator's opinions, feelings, or information about personal experiences associated with data collection or subsequent processing. This study only used data anonymized during the previous data recovery effort, and therefore data used in the analysis for the submitted manuscript could not be linked back to the individual Principal Investigators. Finally, all data used and referenced here are legally public data. No private data were requested or used for this study.
